# Associations of AD Biomarkers and Cognitive Performance with Nutritional Status: The NUDAD Project

**DOI:** 10.3390/nu11051161

**Published:** 2019-05-23

**Authors:** Astrid S. Doorduijn, Marjolein Visser, Ondine van de Rest, Maartje I. Kester, Francisca A. de Leeuw, Sanne Boesveldt, Jay L. P. Fieldhouse, Ellen G. H. M. van den Heuvel, Charlotte E. Teunissen, Philip Scheltens, Wiesje M. van der Flier, Marian A. E. de van der Schueren

**Affiliations:** 1 Department of Nutrition and Dietetics, Amsterdam UMC, Vrije Universiteit Amsterdam, Amsterdam Public Health Research Institute, 1081HV Amsterdam, The Netherlands; m.devanderschueren@amsterdamumc.nl; 2 Alzheimer Center Amsterdam, Department of Neurology, Amsterdam Neuroscience, Amsterdam UMC, Vrije Universiteit Amsterdam, 1081HZ Amsterdam, The Netherlands; m.kester@vumc.nl (M.I.K.); f.deleeuw@vumc.nl (F.A.d.L.); j.fieldhouse@vumc.nl (J.L.P.F.); p.scheltens@vumc.nl (P.S.); wm.vdflier@vumc.nl (W.M.v.d.F.); 3 Department of Health Sciences, Faculty of Science, Vrije Universiteit Amsterdam and the Amsterdam Public Health Research Institute, 1081HV Amsterdam, The Netherlands; m.visser@vu.nl; 4 Division of Human Nutrition and Health, Wageningen University & Research, 6708WE Wageningen, The Netherlands; ondine.vanderest@wur.nl (O.v.d.R.); sanne.boesveldt@wur.nl (S.B.); 5 Neurochemistry Laboratory, Department of Clinical Chemistry, Amsterdam Neuroscience, Amsterdam UMC, Vrije Universiteit Amsterdam, 1081HZ Amsterdam, The Netherlands; c.teunissen@vumc.nl; 6 FrieslandCampina, 3818LE Amersfoort, The Netherlands; ellen.vandenheuvel@frieslandcampina.com; 7 Department of Nutrition and Health, HAN University of Applied Sciences, 6525EJ Nijmegen, The Netherlands

**Keywords:** Alzheimer’s disease, mild cognitive impairment, subjective cognitive decline, malnutrition, older adults, body composition

## Abstract

As malnutrition is common in patients with Alzheimer’s disease (AD), we evaluated nutritional status and body composition of patients with AD, mild cognitive impairment (MCI) and controls, and studied associations of AD biomarkers and cognitive performance with nutritional status and body composition. We included 552 participants, of which 198 patients had AD, 135 patients had MCI and 219 controls. We assessed nutritional status (mini nutritional assessment (MNA)) and body composition (body mass index (BMI), fat-free mass (FFM) and waist circumference). Linear regression analyses (adjusted for age, gender and education where appropriate) were applied to test associations of AD biomarkers and cognitive performance on five domains with nutritional parameters (dependent). Patients with MCI and AD had a lower BMI and MNA score than controls. Worse performance in all cognitive domains was associated with lower MNA score, but not with body composition. AD biomarkers were associated with MNA score, BMI and waist circumference, and associations with MNA score remained after adjustment for cognitive performance. Both AD biomarkers and cognitive performance were associated with nutritional status, associations with AD biomarkers remained after adjustment for cognition. Our data suggest that malnutrition is not only related to impaired cognition but also to AD pathology.

## 1. Introduction

Unintended weight loss and protein energy malnutrition are common features in patients with moderate to severe Alzheimer’s disease (AD) dementia. The prevalence of malnutrition is reported to range from 0%–13% in community-dwelling patients [1,2] to 30%–60% in institutionalized patients [3,4]. Malnutrition in patients with AD is associated with an accelerated progression of disease and increased morbidity and mortality [5,6,7]. Much less investigated is the prevalence of malnutrition in patients with mild cognitive impairment (MCI). The few available studies showed that patients with MCI are at higher risk of malnutrition than cognitively healthy adults, albeit at lower risk than patients with AD [8,9,10]. Population-based studies in non-demented adults found that weight loss is a predictor for incident MCI and dementia [6,11,12], and may be one of the first signs of cognitive problems.

Several hypotheses have been proposed to explain how weight loss is associated with AD. One potential explanation is poor nutritional intake caused by a decline in cognitive functioning. Some examples are forgetting to eat, no longer being able to use eating utensils, chewing problems or dysphagia [13,14]. Second, biological changes in the brain might play a role in changes in the nutritional status of patients with AD [15]. Cerebrospinal fluid (CSF) β-amyloid 42 (Aβ_42_), total tau (tau) and tau phosphorylated at threonine 181 (p-tau) are considered to reflect AD pathophysiology and make it possible to measure AD pathology ‘in vivo’ [16]. AD pathology has been hypothesized to elevate metabolism and therefore energy expenditure, or to alter the uptake of nutrients leading to malnutrition [15]. It has, however, not yet been studied whether AD pathology or cognitive performance in different domains are associated with nutritional status or body composition. The aim of this study was to compare the nutritional status and body composition of patients with AD and MCI to cognitively normal controls. Furthermore, we studied associations of AD biomarkers and cognitive performance with nutritional status and body composition.

## 2. Materials and Methods

### 2.1. Study Population

The NUDAD (Nutrition, the Unrecognized Determinant in Alzheimer’s Disease) study is a prospective cohort studying nutritional determinants in AD and pre-dementia stages, with three-year clinical follow-up. Here, we present cross-sectional baseline data of all participants enrolled in NUDAD. NUDAD is a subsample of the Amsterdam Dementia Cohort, existing of patients who visited our Alzheimer center between September 2015 and August 2017 and were diagnosed with AD, MCI or subjective cognitive decline (SCD) and had a mini-mental state examination (MMSE) score >16 [17]. Patients underwent a standardized dementia screening, including extensive neuropsychological assessment, neurological examination and laboratory tests [18]. Clinical diagnosis of MCI and AD was established by consensus in a multidisciplinary meeting according to the National Institute on Aging-Alzheimer’s Association criteria [19,20]. As a control group, we used subjects with SCD who presented with memory complaints but appeared normal on all clinical examinations, i.e., criteria for MCI, dementia or psychiatric diagnosis were not fulfilled [18]. In total 552 participants were included, 198 patients with AD, 135 patients with MCI and 219 controls. Informed consent was obtained from all participants and the protocol was approved by the Ethics Committee of the Amsterdam UMC(2015.457).

Descriptive characteristics included: age, gender, MMSE score, level of education, living situation (with partner/children, alone, nursing home) and smoking status (current, former, never). Level of education was assessed using the Verhage classification system [21], which we categorized into low (score 1–3), intermediate (score 4 and 5) and high (score 6 and 7). Furthermore, the presence of diabetes mellitus, hypertension, hypercholesterolemia and the history of myocardial infarct were retrieved from medical records. Advanced glycation endproduct (AGE) score, a measure of cardiovascular risk, was measured via skin autofluorescence with the AGE reader [22]. Apolipoprotein E (APOE) ϵ4 status was dichotomized into carrier (1 or 2 ϵ4 alleles) or non-carrier [23].

### 2.2. Body Composition

Body mass index (BMI), also known as the Quetelet Index, was calculated by dividing the measured body weight by the squared measured height (kg/m^2^). Participants were classified as: underweight (BMI < 18.5 kg/m^2^), normal weight (BMI 18.5–24.9 kg/m^2^), overweight (BMI 25–29.9 kg/m^2^), or obese (BMI ≥ 30 kg/m^2^). All circumferences (cm) were measured with a measuring tape in standing position: arm at the mid-upper left arm hanging loosely by the side, calf at the broadest point, waist at the smallest part between the lowest rib and the hip, and the hip at the broadest part [24]. Fat-free mass (FFM, kg) was estimated using multi-frequency bio-electrical impedance analysis (Bodystat Quadscan 4000) and the formula of Kyle [25]. The availability for body composition parameters ranged from 78% for FFM to 100% for BMI.

### 2.3. Nutritional Status

Nutritional status was evaluated with the Mini Nutritional Assessment (MNA) [26,27]. Scores ranged from 0 to 30 with a higher score indicating a better nutritional status. Participants were classified as: malnourished (MNA score <17), at risk of malnutrition (MNA score 17–23.5) or well-nourished (MNA score >23.5) [28]. To avoid that differences in MNA score were driven by differences in cognitive performance, we also analyzed a modified MNA score, leaving out the question about neuropsychological problems. MNA was available in 65% of our study population.

### 2.4. Neuropsychological Assessment

Cognitive performance was measured using a standardized neuropsychological test battery, covering five domains. The domain memory included: total recall on visual association test (VAT) and total immediate and delayed recall of the Dutch version of the Rey auditory verbal learning task [29,30]. The domain attention included: trail making test (TMT) part A, forward condition of digit span, and Stroop test word and color subtasks [31,32,33]. The domain executive functioning included: frontal assessment battery, backward condition of digit span, Stroop test color-word subtask and letter fluency [32,33,34,35]. The domain language included: category fluency (animal naming) and the naming condition of the VAT [29,36]. Finally, the domain visuospatial ability included: dot counting, fragmented letters and number location [37]. Raw test scores were converted into z-scores using the mean and SD of our study population. The test scores for TMT A were log-transformed because they were not normally distributed. Z-scores for TMT A and Stroop were inverted, such that lower scores indicate worse cognitive performance for all cognitive tests. Domain scores were calculated by averaging z-scores of the individual tests within that domain if at least two tests were available. Availability of the domain scores ranged from 93% for visuospatial ability to 98% for memory.

### 2.5. AD Biomarkers

CSF was obtained by lumbar puncture using a 25-gauge needle and collected in 10 mL polypropylene tubes (Sarstedt) following standardized protocols [38]. Aβ_42_, tau and p-tau concentrations were determined with sandwich Innotest ELISAs (Fujirebio, Ghent, Belgium) [39] and available in 393 participants (71%). Aβ_42_ concentrations were adjusted for the drift that occurred over the years [40].

### 2.6. Statistical Analyses

Between-diagnosis group differences in participant characteristics, nutritional status and body composition were tested using analysis of variance (ANOVA) with post-hoc LSD (least significant difference), adjusted t-test for continuous variables and chi-square-test for categorical variables. ANOVAs of nutritional status and body composition were adjusted for age, gender and education. Analyses with FFM were additionally adjusted for height and fat mass. Within the total cohort, we used linear regression analyses to evaluate associations between AD biomarkers in CSF or cognitive domains (independent variables) with nutritional status and body composition variables (dependent variables). All descriptive variables in Table 1 that changed the regression coefficient ≥ 10% were regarded as confounders and included in the model. First, we assessed the association of AD biomarkers with nutritional parameters adjusted for age and gender (Table 3, model 1), followed by additional adjustment for all cognitive domain scores to examine the association of biomarkers independent of cognitive performance (Table 3, model 2). Similarly, the association of cognitive domains with nutritional parameters were adjusted for age, gender and education (Table 4, model 1), followed by additional adjustment for both Aβ_42_ and tau levels to examine the association of cognitive performance independent of AD biomarkers (Table 4, model 2). To test the assumptions of the regression analyses, we plotted and checked residuals of all models, which were all normally distributed. Furthermore, in each model, we checked tolerance values, variance inflation factors and correlations between variables and did not observe multicollinearity. Significance was set at *p* <0.05. All analyses were performed with SPSS version 22 (released 2013, IBM SPSS Statistics for Windows, Armonk, NY, USA).

## 3. Results

Patients with MCI and AD were older, had a lower MMSE score and had a lower level of education than controls (Table 1). Groups did not differ in vascular risk factors. Patients with MCI and AD were more often APOE ϵ4 carriers and had lower Aβ_42_ levels and higher tau and p-tau levels than controls. As expected, cognitive performance on all domains differed between groups, with controls scoring highest and patients with AD scoring lowest.

ANCOVAs of body composition showed that patients with MCI and AD had a lower BMI, and were less likely to be obese than controls (Table 2). In addition, patients with MCI had a lower FFM, adjusted for height and fat mass, compared to patients with AD and controls. Patients with AD had a smaller waist and hip circumference compared to controls. There was no interaction with gender for any nutritional parameter. Groups did not differ in arm or calf circumference. Analyzing nutritional status, both full MNA and modified MNA score differed between groups, with patients with AD scoring lowest. Four patients with AD, one patient with MCI and one control were classified as malnourished. More participants were at risk of malnutrition, 38 patients with AD, 17 patients with MCI and 12 controls (*p* = 0.001).

Adjusted linear regression analyses showed that higher levels of tau and p-tau were related to lower BMI, FFM, waist circumference and lower MNA scores. Lower Aβ_42_ levels were related to lower BMI, waist circumference and full MNA score (Table 3, model 1). In the analyses adjusted for cognitive performance, the association of both tau and p-tau with the full MNA score remained whereas the associations with Aβ_42_ lost significance (Table 3, model 2). Furthermore, the association of p-tau with the modified MNA score remained, while the association of tau with this score lost significance.

Adjusted linear regression analyses of cognitive performance revealed associations of all cognitive domains with the full MNA score, with lower domain scores being related to lower full MNA score (Table 4, model 1). Similarly, poorer performance on the domains attention, executive functioning, language and visuospatial ability, but not memory, was associated with a lower modified MNA score. Additionally adjusted for AD biomarker levels, the association of all cognitive domain scores with full MNA score remained, while the association with the modified MNA remained for the domains executive functioning and visuospatial ability only (Table 4, model 2). There were no associations of cognitive domain scores with BMI, FFM and waist circumference.

## 4. Discussion

The main finding of this study is that lower Aβ_42_ and higher tau and p-tau levels were associated with poorer nutritional status and body composition. Moreover, poorer cognitive performance in all domains was associated with poorer nutritional status, but not with body composition.

Consistent with the literature, patients with AD and MCI had lower BMI and smaller waist and hip circumferences than controls [9,10], while the MNA malnutrition score of patients with MCI was in between the score of controls and patients with AD [8,9,41]. We extend on existing literature by adding the modified MNA score (leaving the question on neuropsychological problems out) showing that patients with AD still scored lower, indicating that differences in MNA score cannot solely be accounted for by neuropsychological problems. These findings are strengthened by the associations of poorer performance in all cognitive domains in relation with lower MNA score, independent of AD biomarker levels. It is conceivable that patients with cognitive impairment forget to eat, which leads to malnutrition. Independent of assessment method, other studies also found that with poorer cognitive performance, the nutritional status is worse as well [42,43,44]. In general, older adults that live alone are at higher risk of malnutrition, however, we did not found an association of living situation with nutritional status in our population [45].

Our results also implicate a biological pathway, since more abnormal AD biomarker levels were associated with lower BMI, waist circumference and MNA score. Even after adjusting for cognitive performance, the associations with the MNA score remained, indicating that malnutrition is also directly related to AD pathology, independent of cognitive decline within AD. The biological connection between AD pathology and malnutrition might translate into an elevated metabolism, due to disease-related specific changes in lipid metabolism, and therefore a higher energy expenditure [15,46]. This needs to be confirmed in future studies in our cohort. Another possible explanation for this biological link is malabsorption of nutrients, which is supported by previous studies showing changes in the microbiome in AD [47,48], also subject for future studies. The downstream processes within the AD pathological cascade seem most important for nutritional status, since more abnormal tau and p-tau levels, but not Aβ_42_, were also associated with lower fat-free mass and lower modified MNA score.

Among the strengths of our study is the availability of both AD biomarkers in CSF and neuropsychological tests in different cognitive domains in a large study population covering participants from the complete AD spectrum. Furthermore, we assessed nutritional status and body composition using a set of concise and objective assessments, and not merely (self-reported) body weight or BMI. This study has some limitations. First, the MNA score was missing in 35% of the study population. However, this percentage was similar across all diagnosis groups and there was no difference in BMI between participants with and without MNA (mean ± SD BMI participants with MNA 25.8 ± 4.1 kg/m^2^, without MNA 26.0 ± 4.1 kg/m^2^; *p* = 0.498) indicating the sample was representative for the study population. Second, the control group consisted of patients diagnosed with subjective cognitive decline (SCD). They visited the clinic because of memory complaints, which were not objectified by extended neuropsychological assessment. Associations might be even stronger if a control group without any cognitive complaints would have been included since persons with SCD are at increased risk of developing AD [49]. Third, we did not have data on physical activity, which might explain differences in FFM. Fourth, this study had a cross-sectional design and therefore causal inferences cannot be established. We cannot exclude that the direction of the associations is different than hypothesized and that, due to reduced dietary intake and therefore poorer nutritional status, cognitive function and biomarker levels are affected. Another option might be that AD biomarkers influence nutritional status, which in turn affects cognitive performance. We are currently following our participants longitudinally with yearly neuropsychological testing and assessment of nutritional status. These longitudinal data will enable us to further investigate the pathways involved.

In conclusion, nutritional status and body composition are poorer in patients with AD compared to controls, and already appear affected in patients with MCI. Both AD biomarkers in CSF and cognitive performance are associated with nutritional status, suggesting that malnutrition and to a lesser extent poorer body composition are not only related to impaired cognition but also directly to AD pathology. These insights advocate monitoring the nutritional status of patients with AD pathology even if they do not yet have severe cognitive impairment.

## Figures and Tables

**Table 1:** Characteristics of the study population according to diagnosis group.

	Controls		MCI		AD		*p*-Value
	*n*		*n*		*n*		
Age (years)	219	60.6 ± 7.7	135	66.3 ± 7.7 ^†^	198	67.4 ± 7.9 ^†^	<0.001
Gender, female	219	103 (47.0)	135	54 (40.0)	198	99 (50.0)	0.193
MMSE score	219	29 (27–29)	135	27 (25–28) ^†^	198	23 (21–25) ^†,‡^	<0.001
Vegetarian dietary pattern	98	10 (10.2)	53	2 (3.8)	74	10 (13.5)	0.191
Level of education							
Low	219	13 (5.9)	135	13 (9.6) ^†^	198	17 (8.6) ^†^	0.014
Intermediate		79 (36.1)		67 (49.6) ^†^		93 (47.0) ^†^	
High		127 (58.0)		55 (40.7) ^†^		88 (44.4) ^†^	
Living situation							
With partner/children	219	163 (74.4)	135	111 (82.2)	198	155 (78.3)	0.223
Alone		56 (25.6)		23 (17.0)		41 (20.7)	
Nursing home		0 (0)		1 (0.7)		2 (1.0)	
Smoking status							
Current	219	28 (12.8)	135	23 (17.0)	198	26 (13.1)	0.759
Former		85 (38.8)		52 (38.5)		73 (36.9)	
Never		106 (48.4)		60 (44.4)		99 (50.0)	
Vascular risk factors							
Diabetes Mellitus	219	16 (7.3)	135	18 (13.3)	198	17 (8.6)	0.151
Hypertension	219	49 (22.4)	135	36 (26.7)	198	52 (26.3)	0.557
Hypercholesterolemia	219	21 (9.6)	135	19 (14.1)	198	29 (14.6)	0.242
Myocardial infarct	219	4 (1.8)	135	7 (5.2)	198	5 (2.5)	0.174
AGE score	175	2.3 ± 0.5	113	2.4 ± 0.6	169	2.4 ± 0.6	0.157
AD biomarkers							
APOE ϵ4 carrier	205	89 (43.4)	129	71 (55.0) ^†^	190	123 (64.7) ^†,‡^	<0.001
CSF Aβ_42_ (pg/mL)	150	1047 ± 298	103	856 ± 321 ^†^	141	592 ± 156 ^†,‡^	<0.001
CSF tau (pg/mL)	149	342 ± 254	103	516 ± 300 ^†^	141	547 ± 433 ^†,‡^	<0.001
CSF p-tau (pg/mL)	149	52 ± 36	103	71 ± 32 ^†^	141	92 ± 37 ^†,‡^	<0.001
Cognitive domain specific z-scores							
Memory	216	0.79 ± 0.56	130	−0.16 ± 0.58 ^†^	193	−0.76 ± 0.57 ^†,‡^	<0.001
Attention	215	0.39 ± 0.58	132	0.05 ± 0.59 ^†^	196	−0.51 ± 0.93 ^†,‡^	<0.001
Executive functioning	215	0.45 ± 0.58	132	0.04 ± 0.54 ^†^	188	−0.61 ± 0.80 ^†,‡^	<0.001
Language	215	0.43 ± 0.60	129	0.08 ± 0.51 ^†^	190	−0.52 ± 0.81 ^†,‡^	<0.001
Visuospatial ability	210	0.30 ± 0.28	127	0.17 ± 0.42 ^†^	175	−0.47 ± 1.11 ^†,‡^	<0.001

Data in mean ± SD; *n* (%); median (interquartile range); AD = Alzheimer’s disease; MCI = mild cognitive impairment; MMSE = mini-mental state examination; AGE = advanced glycation endproduct; APOE = apolipoprotein E; CSF = cerebrospinal fluid; Aβ_42_ = β-amyloid 42; p-tau = phosphorylated tau; ^†^ significantly different from controls upon post-hoc testing; ^‡^ significantly different from MCI upon post-hoc testing.

**Table 2:** Nutritional status and body composition per diagnosis group.

	Controls		MCI		AD		*p*-Value
	*n*		*n*		*n*		
BMI (kg/m^2^)	219	26.7 ± 0.3	135	25.4 ± 0.3 ^†^	198	25.1 ± 0.3 ^†^	0.001
BMI category	219		135		198		<0.001
Underweight (BMI < 18.5)		0 (0)		3 (2.2) ^†^		3 (1.5) ^†^	
Normal weight (BMI 18.5–24.9)		93 (42.5)		58 (43.0) ^†^		111 (56.1) ^†^	
Overweight (BMI 25–29.9)		73 (33.3)		60 (44.4) ^†^		68 (34.3) ^†^	
Obese (BMI ≥ 30)		53 (24.2)		14 (10.4) ^†^		16 (8.1) ^†^	
FFM ^#^ (kg)	181	53.5 ± 0.3	99	51.5 ± 0.4 ^†^	152	52.9 ± 0.3 ^‡^	0.001
Waist circumference (cm)	210	92.3 ± 0.8	127	90.6 ± 0.9	188	89.2 ± 0.8 ^†^	0.024
Hip circumference (cm)	210	102.1 ± 0.6	127	100.9 ± 0.8	188	99.9 ± 0.7 ^†^	0.080
Arm circumference (cm)	210	29.0 ± 0.2	127	28.2 ± 0.3	189	28.4 ± 0.2	0.068
Calf circumference (cm)	210	36.0 ± 0.2	127	35.5 ± 0.3	189	35.8 ± 0.2	0.367
MNA score	133	26.0 ± 0.2	91	25.1 ± 0.3 ^†^	134	24.3 ± 0.2 ^†,‡^	<0.001
Modified MNA score	133	24.3 ± 0.2	91	24.0 ± 0.3	134	23.6 ± 0.2 ^†^	0.050
MNA category	133		91		134		0.001
Malnourished (MNA < 17)		1 (0.8)		1 (1.1)		4 (3.0) ^†^	
At risk of malnutrition (MNA 17–23.5)		12 (9.0)		17 (18.7)		38 (28.4) ^†^	
Well-nourished (MNA > 23.5)		120 (90.2)		73 (80.2)		92 (68.7) ^†^	

Data is presented as mean ± SE; n (%); MCI = mild cognitive impairment; AD = Alzheimer’s disease; BMI = body mass index; FFM ^#^ = fat-free mass, adjusted for height and fat mass; MNA = mini nutritional assessment; all tested using age, gender and education adjusted ANOVA with post-hoc LSD adjusted t-test or chi-square-test; ^†^ significantly different from controls upon post-hoc testing; ^‡^ significantly different from MCI upon post-hoc testing.

**Table 3:** Associations of AD biomarkers in CSF with nutritional status and body composition.

		BMI (kg/m^2^)	FFM (kg)	Waist Circumference (cm)	MNA Score	Modified MNA Score
Aβ_42_	Model 1	0.16 *	0.01	0.17 *	0.22 *	0.11
	Model 2				0.13	0.11
Tau	Model 1	−0.23 *	−0.05 *	−0.14 *	−0.27 *	−0.19 *
	Model 2				−0.15 *	−0.13
P-tau	Model 1	−0.22 *	−0.05 *	−0.14 *	−0.28 *	−0.21 *
	Model 2				−0.17 *	−0.15 *

Data presented as β (regression coefficients); * *p* < 0.05; model 1 adjusted for age and gender; model 2 additionally adjusted for cognitive domain scores (memory, attention, executive functioning, language, visuospatial ability); AD = Alzheimer’s disease; CSF = cerebrospinal fluid; Aβ_42_ = β-amyloid 42; p-tau = phosphorylated tau; BMI = body mass index; FFM = fat-free mass, adjusted for height and fat mass in both models; MNA = mini nutritional assessment.

**Table 4:** Associations of cognitive domains with nutritional status and body composition.

		BMI (kg/m^2^)	FFM (kg)	Waist Circumference (cm)	MNA Score	Modified MNA Score
Memory	Model 1	0.07	0.02	0.05	0.21 *	0.10
	Model 2				0.15 *	0.00
Attention	Model 1	0.01	−0.01	0.00	0.19 *	0.12 *
	Model 2				0.14 *	0.07
Executive functioning	Model 1	0.02	0.01	0.02	0.31 *	0.21 *
	Model 2				0.23 *	0.15 *
Language	Model 1	−0.01	0.00	−0.01	0.18 *	0.11 *
	Model 2				0.18 *	0.12
Visuospatial ability	Model 1	0.06	−0.01	0.07	0.20 *	0.12 *
	Model 2				0.20 *	0.13 *

Data presented as β (regression coefficients); * *p* < 0.05; model 1 adjusted for age, gender and education; model 2 additionally adjusted for Aβ_42_ and tau levels; BMI = body mass index; FFM = fat-free mass, adjusted for height and fat mass in both models; MNA = mini nutritional assessment.
